# Supplementary material for: Patterns of Intron Gain and Loss in Fungi
Source: PLoS Biol. 2004 Nov 30;2(12):e422. doi: 10.1371/journal.pbio.0020422 (PMC532390; doi:10.1371/journal.pbio.0020422)
Supplement: Table S1 — Also available at http://genes.mit.edu/NielsenEtAl/. (4.3 MB ZIP). [file pbio.0020422.st001.zip › NielsenEtAl/html/405.html]

AN6708.1.NCU07659.1.MG09878.1.FG04171.1


```
 CLUSTAL W (1.82) Multiple Sequence Alignments - Introns Inserted


Sequence 1: NCU07659.1	458 aa
Sequence 2: MG09878.1	464 aa
Sequence 3: FG04171.1	456 aa
Sequence 4: AN6708.1	488 aa
Alignment Length: 494 aa
Number Identitical Residues: 243 aa
Alignment Score (without introns) 11301


MG09878.1 	MFAPILRRRALQ-------------------KTSSLR~YVTPA---LTRWYAS--~YPPH
NCU07659.1	MIVPVLSRQALR-------------------HASVAR~VALPS---LTRWYAS--~YPPH
FG04171.1 	MLSAALRRRVLAP------------------THSALR~TGFAAH--VVRHYAS--~FPEH
AN6708.1  	MVASAVRMRVPSASFISKGACSLRRPQASYKFTAAIQ2HQLPALAALSRYYASKS1FPPH
          	*. . :  :.  .:  :..:.:   ..:: .  :  :    .: ::: * ***.: :* *

MG09878.1 	TVVKMPALSPTMTAGNIGAWHKKPGDGIAPGDVLVEIETDKAQMDFEFQEEGVLAKVLKE
NCU07659.1	TVVKMPALSPTMTSGGIGAWQKKPGDKIEPGEVLVEIETDKAQMDFEFQEEGVLAKILKD
FG04171.1 	QVIKMPALSPTMQAGNIGAWQKKIGDSIAPGDVLVEIETDKAQMDFEFQEEGVIAKILKD
AN6708.1  	TIISMPALSPTMTAGNIGAWQKKAGDALQPGDVLVEIETDKAQMDFEFQEEGILAKVLKE
          	 ::.******** :*.****:** ** : **:********************::**:**:

MG09878.1 	TGEKDIAVGN0PIAVLVEEGTDVKAFENFTLADAGGEAPASSPPKEEKNV-EESSKAAST
NCU07659.1	SGEKDVAVGN0PIAILVEEGTDVNAFKDFTLKDAGGETSPAVPKDEPKN---ESTASAPT
FG04171.1 	AGEKDIPVGS0PIAVLVEEGTDVAAFEKFSVEDAGGAAKPAAPKEEKSE-----SKSESA
AN6708.1  	SGEKDVSVGS0PIAVLVEEGTDVAAFESFSLEDAGGEGAGAAPPKETQETPKEAPKASEP
          	:****:.**. ***:******** **:.*:: ****    : * .* .: ...:. :  .

MG09878.1 	PTPTPAPEPENTKSAG-RLQNALDREPNAEPAAKRLANEKGIKLDGVKGSGKNGKITAED
NCU07659.1	PAPTPAPEPENTSFTG-RFQTALEREPNALPAAKRLAREKGIDLRNVKGSGPGGKITEED
FG04171.1 	STPEPSSEPQQYESQG-RLQTALDREPNIAAPAKRLAREKGISIDGIKGTGKNGQITEAD
AN6708.1  	STPQPAAGAYEPDTSGEKLQPSLDREPAISPAAKALALEKGVPIKALKGTGRGGQITKED
          	.:* *:. . : .  *.::* :*:***   ..** ** ***: :  :**:* .*:**  *

MG09878.1 	VKKLGSSGPAAAAAAGPAGALYEDIPISNMRKTIANRLKESVSENPHYFVSSSLSVSKLL
NCU07659.1	VKKALASAPAAGAAA----AAYTDVPISGMRKTIAARLKESVTENPHFFVSTNLSVSKLL
FG04171.1 	VKKAVSSPAASAASS---AASYEDIPISGMRKTIANRLVESTQTNPHFYVTSSLSVSKLL
AN6708.1  	VEKYKPTAAAAAAGP-----ASEDIPLTSMRKTIASRLQQSWNQNPHFFVSTTLSVTKLL
          	*:*  .: .*:.*..        *:*::.****** ** :*   ***::*::.***:***

MG09878.1 	KLRQALNSSSEGKYKLRVNDFLIKAIAVASKKVPQVNSSWRDG----VIRQFNTVDVSVA
NCU07659.1	KLRQALNSSADGRYKLSVNDFLIKAMGIASKRVPTVNSSWRDG----VIRQFETVDVSVA
FG04171.1 	KLRQALNASADGKYKLSVNDFLIKAIAVASRKVPQVNSSWRDG----NIRQFNTVDVSVA
AN6708.1  	KLRQALNASSEGKYKLSVNDFLIKACAAALRKVPQVNSSWTEENGQVVIRQHNSVDISVA
          	*******:*::*:*** ******** . * ::** ***** : ...  ***.::**:***

MG09878.1 	VSTPSGLITPIVKAVETKGLESISASVKELAKRARDNKLKSDEYQGGTISISNMGMNTAV
NCU07659.1	VATPNGLITPIVKGVEGKGLESISAAVKELAKKARDGKLKPEEYQGGSISISNMGMNPAV
FG04171.1 	VSTPTGLITPIVTGVEGRGLEAISAQVKSLAKKARDGKLKPEEYQGGTISISNMGMNPAV
AN6708.1  	VATPVGLITPIVKNAQGLGLSSISNQVKDLGKRARDNKLKPEEYQGGTFTISNMGMNPAV
          	*:** *******. .:  **.:**  **.*.*:***.***.:*****:::*******.**

MG09878.1 	ERFTAVINPPQAAIVAIGTTKKVAVPVENEDGTTGVEWDDQIVITGSFDHKVVDGAVGAE
NCU07659.1	QSFTAIINPPQAAILAVGAPQKVAVPVENEDGTTGVSWDEQIIVTASFDHKVVDGAVGAE
FG04171.1 	DHFTAVINPPQAAILAVGTTKKVAIPSDNE---AGVEFDDQITLTASFDHKVVDGAVGAE
AN6708.1  	ERFTAIINPPQAGILAVGTTRKVAVPVETEEG-TSVEWDDQIIVTASFDHRVVDGAVGAE
          	: ***:******.*:*:*:.:***:* :.*.. :.*.:*:** :*.****:*********

MG09878.1 	WMKELKTVVENPLQLLL
NCU07659.1	WIRELKKVIENPLELLL
FG04171.1 	WLKEVKKVIENPLELLL
AN6708.1  	WIKELKKVVENPLELLL
          	*::*:*.*:****:***
```
